# First Human Rabies Case in French Guiana, 2008: Epidemiological Investigation and Control

**DOI:** 10.1371/journal.pntd.0001537

**Published:** 2012-02-21

**Authors:** Jean-Baptiste Meynard, Claude Flamand, Céline Dupuy, Aba Mahamat, Françoise Eltges, Frederic Queuche, Julien Renner, Jean-Michel Fontanella, Didier Hommel, Philippe Dussart, Claire Grangier, Félix Djossou, Laurent Dacheux, Maryvonne Goudal, Franck Berger, Vanessa Ardillon, Nicolas Krieger, Hervé Bourhy, André Spiegel

**Affiliations:** 1 Institut Pasteur de la Guyane, Cayenne, French Guiana; 2 Regional Epidemiology Cell, Cayenne, French Guiana; 3 Departmental Veterinary Direction, Cayenne, French Guiana; 4 Centre Hospitalier Andrée-Rosemond, Cayenne, French Guiana; 5 Health Regional Agency, Cayenne, French Guiana; 6 Institut Pasteur, Centre National de Référence pour la Rage, Paris, France; Yale University, United States of America

## Abstract

**Background:**

Until 2008, human rabies had never been reported in French Guiana. On 28 May 2008, the French National Reference Center for Rabies (Institut Pasteur, Paris) confirmed the rabies diagnosis, based on hemi-nested polymerase chain reaction on skin biopsy and saliva specimens from a Guianan, who had never travelled overseas and died in Cayenne after presenting clinically typical meningoencephalitis.

**Methodology/Principal Findings:**

Molecular typing of the virus identified a *Lyssavirus* (*Rabies virus* species), closely related to those circulating in hematophagous bats (mainly *Desmodus rotundus*) in Latin America. A multidisciplinary Crisis Unit was activated. Its objectives were to implement an epidemiological investigation and a veterinary survey, to provide control measures and establish a communications program. The origin of the contamination was not formally established, but was probably linked to a bat bite based on the virus type isolated. After confirming exposure of 90 persons, they were vaccinated against rabies: 42 from the case's entourage and 48 healthcare workers. To handle that emergence and the local population's increased demand to be vaccinated, a specific communications program was established using several media: television, newspaper, radio.

**Conclusion/Significance:**

This episode, occurring in the context of a Department far from continental France, strongly affected the local population, healthcare workers and authorities, and the management team faced intense pressure. This observation confirms that the risk of contracting rabies in French Guiana is real, with consequences for population educational program, control measures, medical diagnosis and post-exposure prophylaxis.

## Author Summary

Until 2008, rabies had never been described within the French Guianan human population. Emergence of the first case in May 2008 in this French Overseas Department represented a public health event that markedly affected the local population, healthcare workers and public health authorities. The antirabies clinic of French Guiana, located at Institut Pasteur de la Guyane, had to reorganize its functioning to handle the dramatically increased demand for vaccination. A rigorous epidemiological investigation and a veterinary study were conducted to identify the contamination source, probably linked to a bat bite, and the exposed population. Communication was a key factor to controlling this episode and changing the local perception of this formerly neglected disease. Because similar clinical cases had previously been described, without having been diagnosed, medical practices must be adapted and the rabies virus should be sought more systematically in similarly presenting cases. Sharing this experience could be useful for other countries that might someday have to manage such an emergence.

## Introduction

Worldwide, rabies causes approximately 55,000 deaths per year [1]. Rabies viruses are transmitted to humans via saliva from bites of carnivores and bats. Bats may be frugivorous, hematophagous or insectivorous. Vampire bats (3 main species *Desmodus rotundus*, *Diphylla ecaudata* and *Diaemus youngi*) feed on blood from warm-blooded animals, e.g. horses and cattle [2].

Rabies in 2005, transmitted to humans by vampire bats reached new heights in Latin America, where with several outbreaks reportedly concerned 55 human cases, 41 of them in the Amazon region of Brazil. Peru and Brazil had the highest numbers of reported cases from 1975 to 2006 [3]. Bats represent the main vector of human rabies in Brazil [4]–[8]. Near its border with French Guiana, other outbreaks were described in remote rural areas of Portel and Viseu Municipalities, Pará State, northern Brazil. Twenty-one human deaths were attributed to paralytic rabies in those 2 municipalities. Isolates were antigenically characterized as *D. rotundus* variant 3 [9]. During a recent outbreak, media reports noted that nocturnal biting coincided with the failure of a regional generator that left people without electricity for 6 weeks. Outbreaks of bat-transmitted rabies have been linked to the continued deforestation of the Amazon region, which has displaced vampire bats across northern Brazil and increased their contact with humans. The reasons for the outbreak in Brazil are not yet fully understood.

In French Guiana, a French Overseas Department located in South America, 10 cows, 2 dogs and 1 cat died of bat rabies-virus infection between 1984 and 2003 [10], [11], but no human case had previously been reported there [10]–[12]. However, on 28 May 2008, the National Reference Center for Rabies (Institut Pasteur, Paris), confirmed the diagnosis of rabies for a 42-year-old French Guianan man, who had never left this Department and who died in Cayenne, after developing clinically typical meningoencephalitis. Since 14 May, he had complained of nonspecific symptoms, mainly fever, severe asthenia and pain, and had consulted at the Cayenne Hospital Emergency Unit 3 times before being admitted on 21 May in a state of mental confusion; his condition deteriorated rapidly thereafter. He became comatose on the same day and died on 27 May. On 28 May, rabies was diagnosed based on a new reverse-transcription hemi-nested polymerase chain reaction (RT-hnPCR) protocol applied to a skin biopsy and saliva specimens. This case illustrates the risk of under-reporting of human rabies based only on clinical criteria and highlights the need for laboratory confirmation to obtain accurate data on disease burden [13]–[15]. Phylogenetic analysis of the isolated virus identified a *Lyssavirus* (*Rabies virus* species), closely related to those circulating in hematophagous bats. This identification of the first human case of bat rabies in France resulted in the creation of a national multidisciplinary Crisis Unit under the authority of the French Ministry of Health in Paris. In French Guiana, it was coordinated by the local health authorities and the Center for Treatment Anti-Rabies (CTAR) of Institut Pasteur de la Guyane (IPG). Its objectives were to manage the crisis, implement an epidemiological investigation and a veterinary survey, provide control measures and establish a communications program. Herein, we review the methodology used by the Crisis Unit and the consequences of this case on the local perception of rabies.

## Methods

### Crisis Unit

Immediately following laboratory confirmation of the rabies case, a multidisciplinary Crisis Unit was created at the national level in Paris, France, and locally in Cayenne, Guiana. Nationally, the stakeholders were affiliated with the French Ministry of Health, French Ministry of Agriculture, Institut Pasteur, Paris, and Institut de Veille Sanitaire, St-Maurice. Locally, the involved services were IPG and its CTAR, Departmental Health and Development Direction, Departmental Veterinary Services, Cayenne Hospital and the Regional Epidemiological Cell.

### Epidemiological investigation and control within exposed population

An epidemiological investigation was conducted to identify people potentially exposed to rabies virus, who would require post-exposure prophylaxis (PEP). To do so, the following criteria of exposure were used. A person was defined as potentially exposed when: 1) he/she was a part of the case's entourage (family, friends, sexual partners, sport team members, colleagues, visitors) during the 15 days preceding the onset of the index case's symptoms; 2) he/she was a healthcare worker who had cared for the case; or 3) he/she had been in contact with animals suspected of being contaminated (based on their behavior, illness, death) that were known to have been in contact with the case [16]. An active search was carried out within the case's familial and professional entourage, and in the Cayenne hospital, where the patient had been admitted. A step-by-step search procedure was implemented, questioning all exposed people able to identify other people suspected of being in contact with the index case. All people suspected of exposure had a medical visit at the IPG CTAR, using Questionnaire 2 (Table S2) for the case's familial and professional entourage.

For the healthcare workers, the exposure level was assessed for those working in 3 of Cayenne Hospital's departments: emergency unit, intensive care unit and biology laboratories. To assess their possible exposure, specific healthcare worker questionnaire 1 (Table S1) was filled out during a medical consultation with the physician responsible for the hospital's hygiene unit.

A healthcare worker was considered to be exposed when: 1) he/she had close contact with the index case (<1 m), took part in his resuscitation or performed an act susceptible of generating aerosolization of body fluids; 2) he/she had close contact with the case's biological fluids (laboratory workers); 3) he/she had been bitten by the case; or 4) he/she failed to comply with general hygiene recommendations.

When exposure corresponded to those criteria, the 4-dose Zagreb immunization protocol (1 of the schedules recommended by the World Health Organization guidelines [1]) against rabies was systematically administered.

Chi-square or Fisher's exact test, with a risk α of 5% was used for simple comparisons of rates. The statistical analyses were run with SAS version 9.1 (SAS Institute Inc., Cary, NC, USA).

### Veterinary survey

The rabies index case had numerous contacts with animals. The objectives of the veterinary survey were to identify the animal at the origin of the rabies infection and to identify other animals possibly contaminated and currently in the incubation period. Suspected animals were listed by questioning the case's family and close contacts. Standardized veterinary questionnaire 3 (Table S3) was completed at IPG, when people in close contact with the case came for their vaccinations, in collaboration with the Regional Epidemiological Cell and the Departmental Veterinary Services.

Two types of suspected animals were identified: those still alive and those that had died. Although it was impossible to obtain brain samples from most of the deceased animals, some of their graves could be found and sometimes brain specimens could be recovered. The living suspected animals were placed under veterinary surveillance and some of them were euthanized when suspicious clinical symptoms became manifest. All the animal brain samples were sent to the National Reference Center for Rabies for laboratory diagnosis of rabies.

### Communications

The communications program had several levels (national and local) and targeted different types of people (general population, healthcare workers and veterinary services). Several communication means were used: national and local press releases, press conferences, and specific meetings with healthcare workers (in Cayenne Hospital and within the private-practice network) and veterinarians in private practice. The local media (television, radio, print) were kept informed and recruited to encourage individuals who might have been in contact with the index case to come and consult at the IPG CTAR.

## Results

### Crisis Unit

**Figure 1:**
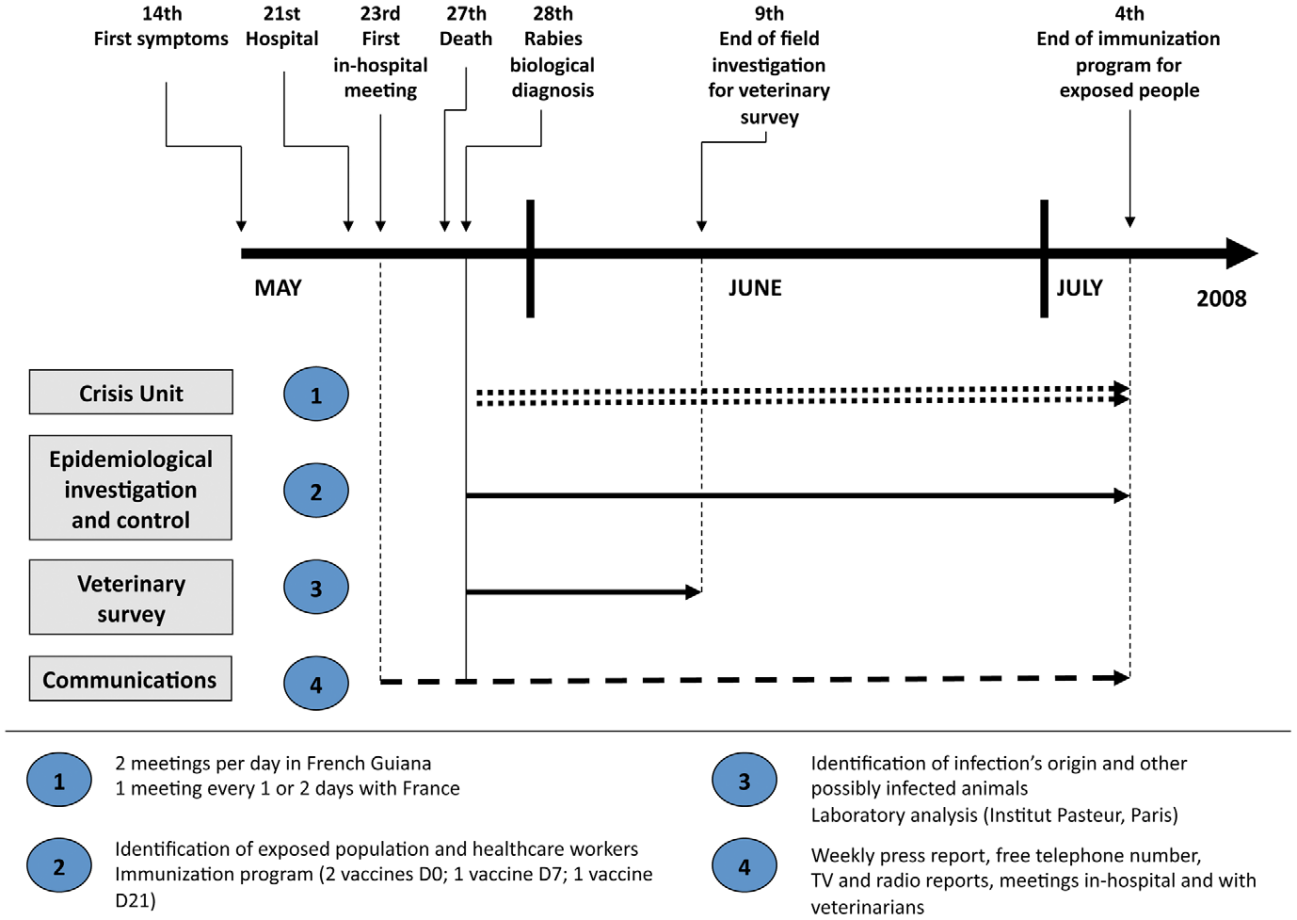
Time schedule of the main measures taken after emergence of the rabies case.

The Crisis Unit met twice daily in French Guiana from 28 May 2008 to 4 July 2008 (Fig. 1). The stakeholders involved in continental France participated by phone once daily until mid-June then every 2 days thereafter. After each meeting, a brief report was sent to all the stakeholders to share up-to-date information with the entire network of people involved in the epidemiological investigation, control measures and the veterinary survey. It also provided regular updates of the situation to the media and, thus, to the local population.

### Epidemiological investigation and control within exposed population

**Figure 2:**
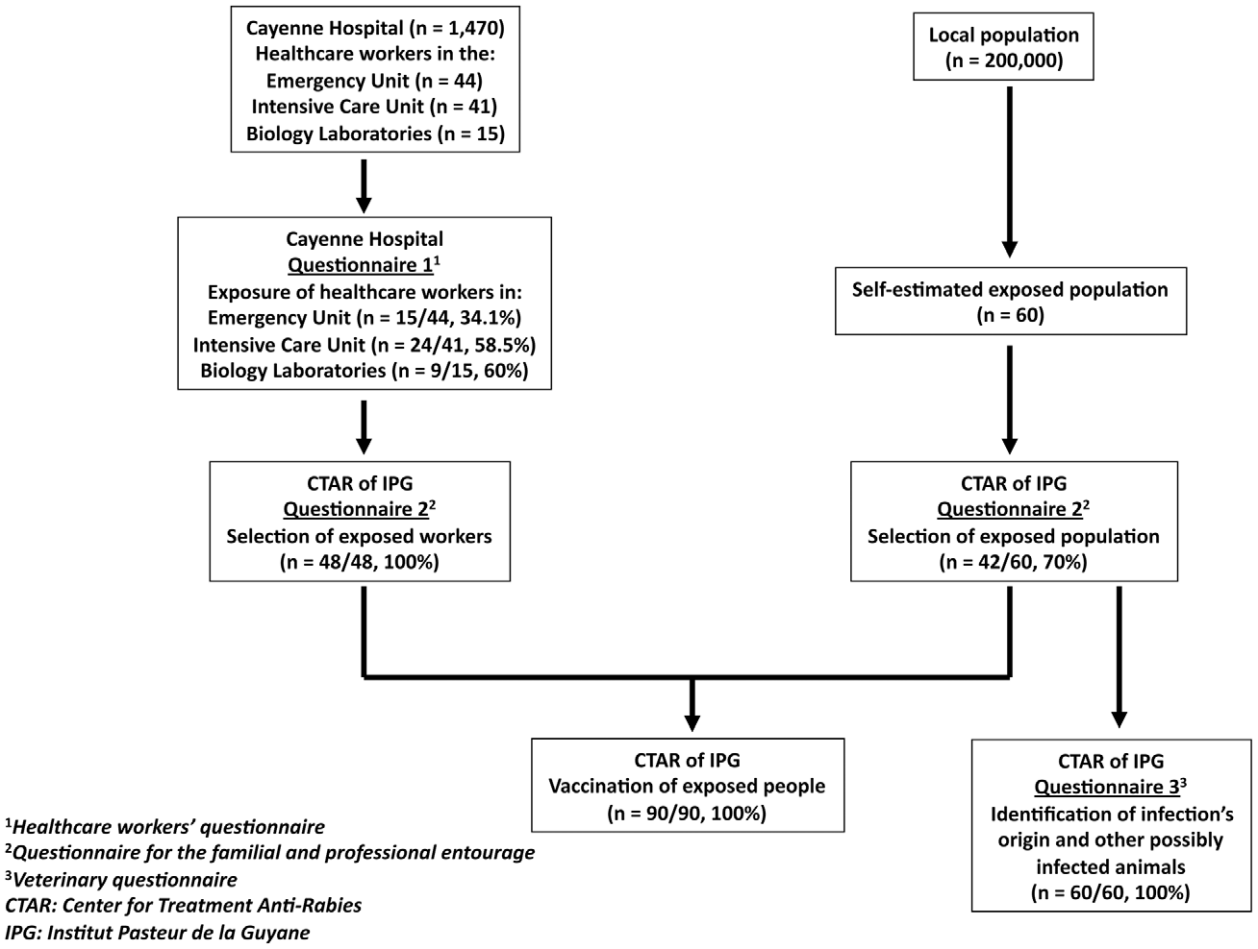
Flow diagram of the procedure for selecting people requiring post-exposure prophylaxis.

**Figure 3:**
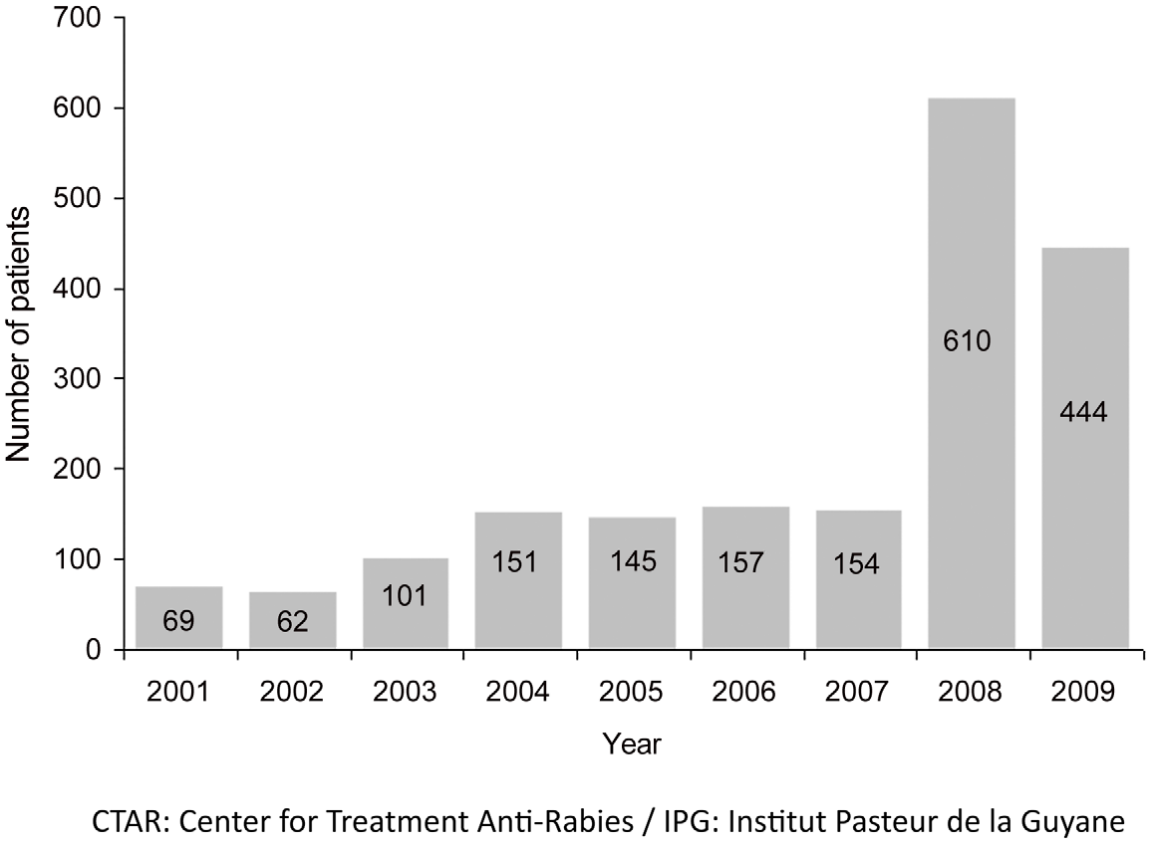
Number of patients consulting at the CTAR of IPG, 2001–2009.

This investigation identified 160 persons suspected of being exposed: 60 within the index case's entourage and 100 in Cayenne Hospital (Fig. 2, Table 1). Each one's risk of exposure was systematically assessed by the CTAR, after an individual interview with a doctor. Exposure was confirmed for 90 persons: 42/60 (70%) from the case's entourage and 48/100 (48%) from the hospital. The first medical interview conducted within the hospital identified 48 healthcare workers; the CTAR confirmed all of them. For healthcare workers, the confirmed exposure rate differed among departments: 60% for biology laboratories, 58.5% for the intensive care unit and 34.1% for the emergency unit (*p* = 0.04), and significantly so between the former 2 (*p* = 0.01) and the latter 2 (*p* = 0.02). All 90 exposed individuals were vaccinated against rabies. The immunization program for these people began between 28 May and 13 June, and finished on 4 July. Because reported exposures were considered to be only grade 2, no rabies immunoglobulins were administered. One of the consequences of human rabies emergence was a higher number of consultations at the CTAR (Fig. 3), necessitating the reorganization of its functioning and establishment of dedicated chain of consultation for rabies [17].

**Table 1:** Distribution of exposed individuals, and those vaccinated within the rabies case's contacts, French Guiana, 2008.

	Exposed individuals		No. (%)^2^ of persons
Nature of contact	No. suspected	No. (%)^1^ confirmed	vaccinated
Index case's entourage	60	42 (70)	42 (100)
Contact with the index case	49	36 (84.7)	36 (100)
Sexual contact, exchange of saliva	13	12 (92.3)	12 (100)
Participated in case care	15	14 (93.3)	14 (100)
Other	21	10 (46.6)	10 (100)
Contact with suspected animal	11	6 (54.5)	6 (100)
Scratches	1	1 (100)	1 (100)
Other	10	5 (50)	5 (100)
Healthcare workers	100	48 (48)	48 (100)
Medical specialists	8	6 (75)	6 (100)
General practitioners	8	5 (62.5)	5 (100)
Nurses	38	19 (50)	19 (100)
Nurse's aides	15	7 (46.7)	7 (100)
Laboratory technicians	13	8 (61.6)	8 (100)
Housekeeping personnel	8	1 (12.5)	1 (100)
Radiology technicians	8	2 (25)	2 (100)
Trainees	2	0	0 (100)
Total	160	90 (56.3)	90 (100)

1 Number of confirmed exposed people/number of suspected exposed persons.

2 Number of immunized people/number of confirmed exposed persons.

### Veterinary survey

Exposure of the rabies index case to bats was difficult to assess. He had very frequently slept outside, in a hammock but without using mosquito netting to protect him, in places where bats are numerous. Nobody from his entourage recalled his having been bitten by a bat but, because bats usually bite while its victim is sleeping, it is impossible to formally conclude. For other animals, a field investigation was conducted from 29 May to 9 June to identify retrospectively any animal that might have been the source of contamination of the human case. Five suspected pets were identified: 4 cats and 1 dog. Unfortunately, only 1 exploitable carcass was recovered and it yielded negative results (Table 2). Four other cats living in the index case's residence, in contact with those pets suspected of having been exposed to the rabies virus, were placed under veterinary observation and, finally, euthanized on 30 May 2008. All the biological analyses were negative. This survey was unable to identify the source of rabies transmission.

**Table 2:** Chronology of potentially at-risk rabies exposures listed for the rabies case, French Guiana, 2008.

	March 2007	January 2008	March 2008	9 May 2008	Before 10 May 2008
Suspected animal	Kitten found dead in a pool of blood at the index case's residence	Wandering cat considered aggressive by neighbors	Cat living in the index case's residence	Cat living in the index case's residence found inanimate	Dog living in the index case's residence found dead (10 May): no distinguishing features
Type of contact	Attempted resuscitation of the animal	Bitten after handling the cat and attempting care	Bitten during administration of veterinarian- prescribed treatment	Mouth-to-mouth resuscitation attempted	Simple physical contact (petting…)
Animal's outcome	Dead & buried in the garden	Animal disappeared after biting	Animal disappeared after biting	Cured after intensive care	Dead & buried in the garden
Measures taken	None	None	None	Euthanized for analysis (30 May): negative results	Exhumed for analysis, samplesnot usable

### Communications

A local press conference was organized each week until mid-June. Three reports, on the epidemiological investigation, control measures and veterinary survey, were specifically written for the print media. A free telephone number, dedicated to questions about rabies, was opened within the local Health Authorities Services. Four television and radio reports were prepared within the IPG CTAR.

Three meetings were organized at Cayenne Hospital to inform healthcare workers. The first was held on 23 May, before rabies had been biologically confirmed, to anticipate the crisis and assure the medical teams' preparedness to react (Fig. 1). It was useful because it enabled a quicker healthcare-worker response when the rabies diagnosis was confirmed. Another meeting was held on 2 June to inform private-practice veterinarians in French Guiana of the epidemiological developments and their role in the ongoing epidemiological survey.

## Discussion

The last autochthonous human rabies case identified any French territory was reported in 1924 in continental France [11]. However, the risk remains of humans being exposed to the virus in enzootic countries and not seeking PEP due to ignorance of the rabies risk. Since 1970, 21 human deaths from rabies have been recorded in France [18]: 20 cases were imported and 1 was transmitted by a corneal transplant. The first human rabies case diagnosed in French Guiana, in May 2008, and described herein, confirms that the risk of contracting the rabies virus there indeed exists. It was the first case subjected to molecular biology confirmation by the French National Reference Center for Rabies.

The patient's initial clinical picture was not typical and he consulted the Cayenne Hospital emergency unit 3 times before being admitted, further emphasizing the need to include rabies in the differential diagnosis of unexplained encephalitis in humans [14]. Rabies was diagnosed intravitam based on RT-hnPCR–detection of viral RNA in saliva and a skin biopsy [13]. The rabies virus responsible was similar to those circulating in hematophagous bats in this part of the world and closely related to those previously isolated from animals in French Guiana with <4% nucleotide divergence in the nucleoprotein gene (unpublished data). The origin of the contamination was not formally established, although an unrecognized vampire-bat bite seems by far the most likely route of transmission. However, as some cases reported in other countries [19], the source of contamination could also have been feline, because a cat reportedly died in March 2008, 2 months after having been severely bitten and wounded by a bat.

After the public was informed of this case, the number of patients consulting the CTAR increased dramatically [17], a phenomenon that had previously been observed in continental France [20]. Since 2008, no other human rabies case has been reported in French Guiana.

Recent emerging zoonoses, e.g., Ebola or Marburg virus hemorrhagic fevers, Nipah virus encephalitis, severe acute respiratory syndrome (SARS), highlight the potential of bats as vectors for transmission of infectious diseases to humans. This potential was already known for rabies encephalitis, since 10 of the 11 *Lyssavirus* species are transmitted by bats. Rabies control in bats remains very difficult, even though some encouraging experimental results obtained with *D. rotundus* bats in captivity demonstrated the immunogenicity of the vaccinia-rabies glycoprotein [21]. However, several effective methods are available to limit the access of the bat population to cattle. Furthermore, some preventive and control measures to limit the number of human deaths attributable to rabies transmitted by vampire bats have been successfully implemented [3].

Rabies diagnosis is a key issue. It is routinely based on clinical and epidemiological information, especially when the exposure is reported in a rabies-endemic country. Although techniques for postmortem diagnosis of rabies have been well-established for decades, tests for *intravitam* diagnosis of human rabies were rarely optimal, and depended entirely on the nature and quality of the sample supplied. Over the past 3 decades, molecular biology tools have contributed to the development of these tests, resulting in more rapid detection of the rabies virus. Several molecular methods are now available that can be used to complement conventional tests for human rabies diagnosis [22]. The 21^st^ century challenges for diagnostic test developers are 2-fold: first, to achieve internationally accepted validation of a test that will then lead to its acceptance by international organizations; second, these tests are mainly needed in developing regions the world, where financial and logistical barriers prevent their implementation [14], [22]. The question is even more important in that rapid diagnosis of rabies in suspected human cases influences PEP for potential case contacts and ensures appropriate patient management [23].

This first human rabies case in French Guiana means that national and local public health authorities must improve preventive and control measures for the local population and travellers. Rabies prophylaxis requires a multifaceted approach, including health education, PEP, systematic vaccination of dogs and cats, and, sometimes, selective immunization campaigns to control transmission among wild animals, e.g. foxes and hematophagous bats [24]. Since human rabies is almost always fatal if prophylactic measures are not initiated, it is essential to increase awareness of who should receive PEP and when it should be administered.

Pre-exposure prophylaxis entails the administration of the rabies vaccine to individuals at high risk for exposure to rabies viruses, e.g., laboratory workers who handle infected specimens, diagnosticians, veterinarians, animal-control workers, rabies researchers, cave explorers… [25].

PEP consists of a multimodal approach to decrease an individual's likelihood of developing clinical rabies after suspected exposure to the virus. Regimens depend on the victim's vaccination status and involve a combination of wound cleansing, rabies-vaccine inoculation, and administration of human rabies immunoglobulins [25]. When used in a timely and accurate fashion, PEP is nearly 100% effective. However, once clinical rabies manifestations have developed, rabies PEP remains supportive. To date, only 5 well-documented cases of prolonged survival or recovery from rabies have been described and were specifically associated with PEP administration before the onset of symptoms [26]. The recently developed Milwaukee protocol added induction of therapeutic coma to supportive care measures and antivirals, claiming it ensured the recovery of an unvaccinated patient. However, its use has yielded inconsistent outcomes [27].

The impact of this rabies-virus emergence in French Guiana was dramatic, especially in the context of a Department far from continental France. Despite the enormous pressure placed on the crisis-managing team by the local population, healthcare workers and politicians, the number of PEP remained relatively limited compared with previous cases in continental France [16] and other countries [28]–[32]. Notably, no subsequent case developed.

This case illustrates the need for further preparedness of public health infrastructures in rabies-enzootic areas that have not yet recorded human rabies cases. Pertinently, lessons learned from other countries, informing public health professionals and a multidisciplinary approach were essential to crisis management of our case [3], [33]. His case history enhanced the perception of the risk and, consequently, a vast campaign to educate and inform the general population about zoonotic diseases acquired from domestic, as well as wild animals, like bats, was undertaken in French Guiana as had been done in neighboring countries [34]. In addition to these measures, rabies is now more systematically included in the differential diagnosis of human encephalitis cases consulting at French Guiana hospitals. Indeed, 2 suspected human cases, subsequently found negative, were subjected to rabies testing during 2008–2010 period. In parallel, active surveillance of bat rabies has been established to learn more about rabies-virus circulation in the local bat populations.

## Supporting Information

Table S1 Questionnaire 1 – Exposure of Healthcare Workers.
(PDF)

Table S2 Questionnaire 2 – Evaluation of the risk of exposure to rabies by the case's entourage.
(PDF)

Table S3 Questionnaire 3 – Veterinary contact with rabies.
(PDF)
