# Supplementary material for: First Human Rabies Case in French Guiana, 2008: Epidemiological Investigation and Control
Source: PLoS Negl Trop Dis. 2012 Feb 21;6(2):e1537. doi: 10.1371/journal.pntd.0001537 (PMC3283561; doi:10.1371/journal.pntd.0001537)
Supplement: Table S1 — Questionnaire 1 – Exposure of Healthcare Workers. (PDF) [file pntd.0001537.s001.pdf]

QUESTIONNAIRE 1: Exposure of Healthcare Workers

~~~~~~

Family name:______________________________ First name:___________________________

Position:__________________________ Department:____________________________

Day at work: □14/05/08 □19/05/08 □20/05/08 □other:

Working hours: □7–14 h □14–21 h □21–7 h

Search for potential exposure:

Contact <1 m: □Yes □No

Patient interviewed: □Yes □No

Buccal examination: □Yes □No

Orotracheal intubation: □Yes □No

Tracheal aspiration: □Yes □No

Aerosol therapy: □Yes □No

Respiratory physiotherapy: □Yes □No

Bronchial endoscopy: □Yes □No

Projection of biological fluids: □Yes □No

Lumbar puncture: □Yes □No

Nursing care: □Yes □No

Treatments of the mouth: □Yes □No

Contact patient with fluid(s) and/or mucosa: □Yes □No

Bitten by the patient: □Yes □No

Contact: □No □blood □urine □feces □CSF

□ Handled biological specimens in the laboratory

Application of standard precautions:

Wearing of: □No □gloves □surgical mask

□ lab coat or apron □glasses

□ Handled tubes containing biological specimens under the hood

Exposure to rabies risk:

Probable exposure: □ Yes □ No

Medical management by the Center for Treatment Anti-Rabies of the Institut Pasteur de la Guyane:

Person to be seen again: □ Yes □ No
~~~~~~
