# Supplementary material for: First Human Rabies Case in French Guiana, 2008: Epidemiological Investigation and Control
Source: PLoS Negl Trop Dis. 2012 Feb 21;6(2):e1537. doi: 10.1371/journal.pntd.0001537 (PMC3283561; doi:10.1371/journal.pntd.0001537)
Supplement: Table S2 — Questionnaire 2 – Evaluation of the risk of exposure to rabies by the case's entourage. (PDF) [file pntd.0001537.s002.pdf]

Questionnaire 2: Information sheet: Evaluation of the risk of exposure to rabies by the case's entourage

Identifying no.
|__|__|-|__|__|__|
do not complete

Requested by

Facility: .... **Telephone**: ....
Doctor: ....
Date of consultation: |__| |__| / |__| |__| / |__| |__|

Patient

Family name: ……………………………………………… **First name**: ……………………………………
Birth date: |__| |__| / |__| |__| / |__| |__| Sex: ❑ M   ❑ F

<u>Contact information</u>

Street:………………………………………………… District: …………………………………………......
City: ………………………………County: ………………………Postal code: …………………..
Country: ………………………………Telephone (land line/mobile): ……………… /……………………

<u>Vaccination history</u>: ❑ Yes ❑ No

If Yes: Date of last vaccination: |__| |__| / |__| |__| / |__| |__| Place: …………………………...
Type of vaccine used: ……………………………………………………
Previous allergic reactions to vaccination: ❑ Yes ❑ No
If Yes, which reaction(s) to which vaccine(s) ? …………………………………………………………..

<u>Current episode of exposure to rabies</u>

Date of exposure: |__| |__| / |__| |__| / |__| |__| Approximate time: …………..………………
Site of exposure: District: ……………………………..City: …………………………..…………
County: ………………………………………… Country: ……………………………………….…...

Exposure type 1: contact with the rabies index case

❑ Contact <1 m from the patient
❑ Bitten by the patient   ❑ Resuscitation manoeuvres, especially respiratory
❑ Participation in act generating aerosolization of the patient's respiratory secretions (aerosol therapy, tracheal aspiration, bronchial and/or digestive endoscopy, respiratory physiotherapy, endotracheal intubation and aspiration)
❑ Laboratory personnel in contact with biological fluids
Anatomical Site: ………………………...
Number: ❑ Single ❑ Multiple   If multiple, how many: ………………………………

Exposure type 2: contact with a suspicious animal in the index case's entourage

❑ Bite ❑ Scratch ❑ Licked ❑ Contact
Anatomical site(s):
…………………………………………...
Number: ❑ Single ❑ Multiple   If multiple, how many: ………………………………
Severity: ❑ Deep ❑ Superficial

General treatment of the current episode of rabies exposure

Local disinfection: ❑ Yes ❑ No Type: ……………………………………………………

Sutures: ❑ Yes ❑ No Number: ……………………………………………

Antibiotics: ❑ Yes ❑ No Molecule: ...………………………………………

Anti-tetanus vaccination: ❑ Current ❑ To be done

Specific treatment of the current episode of rabies exposure

1st injection: date: l__l l__l / l__l l__l / l__l l__l Batch #:……………….. Reaction: ……………………

2nd injection: date: l__l l__l / l__l l__l / l__l l__l Batch #:……………….. Reaction: ……………………

3rd injection: date: l__l l__l / l__l l__l / l__l l__l Batch #:……………….. Reaction: ……………………

Protocol completed: ❑ Yes ❑ No

If No, reason for stopping: ❑ Medical decision (risk excluded by veterinarian) ❑ Lost-to-follow-up

Immunoglobulins: ❑ Yes ❑ No Name of product: …………………………………

Site(s) of immunoglobulin injection(s): ………………………….…………………………………

Dose(s) of immunoglobulins injected: ……………………………………………………………..

Animal

Animal species at the origin of the exposure:

❑ Dog ❑ Cat ❑ Bat ❑ Other

If another, species responsible: ……………………………………………………………………

Rabies vaccine status: ❑ Vaccinated ❑ Not vaccinated ❑ Unknown

Date of validity of the anti-rabies vaccination: l__l l__l / l__l l__l / l__l l__l

Owner: ❑ Known ❑ Unknown

Family name of Owner: ………………………………… First name: …………………………………..

Owner's address: Street: ……………………………………District: ……………………………

City: …………………………….. County: ……………….……….. Country: ………………...……...

Telephone (land line/mobile): ……………………………/……………………………………………

Veterinary examination to be scheduled: ❑ Yes ❑ No

1st visit: date: l__l l__l / l__l l__l / l__l l__l Result: ……………………………………………..

2nd visit: date: l__l l__l / l__l l__l / l__l l__l Result: ……………………………………………..

3rd visit: date: l__l l__l / l__l l__l / l__l l__l Result: ……………………………………………..

Laboratory tests: ❑ Yes ❑ No Result: ……………………………………………..

Remarks

Diverse comments:

Date form completed: l__l l__l / l__l l__l / l__l l__l

Doctor's signature

…………………………………………
