# Supplementary material for: First Human Rabies Case in French Guiana, 2008: Epidemiological Investigation and Control
Source: PLoS Negl Trop Dis. 2012 Feb 21;6(2):e1537. doi: 10.1371/journal.pntd.0001537 (PMC3283561; doi:10.1371/journal.pntd.0001537)
Supplement: Table S3 — Questionnaire 3 – Veterinary contact with rabies. (PDF) [file pntd.0001537.s003.pdf]

Questionnaire 3: Veterinary contact with rabies

Number CONTACT /____ **Date of inquiry** . . / . . / . . . . **Interviewer**:...................

Family name: .... **First name**: ....

Address: ....

Sex M ☐ F ☐ **Birth date**: . . / . . / . . . .

Type de contact suspected: Animal ☐ **Human** ☐ **Where contact occurred:.....................**

Contact animal	Date of contact	Type of contact[1]					
Ribouc (dog)	between 25/04 & 10/05	☐	B	S	L	Tx	SPC
Grey cat	between 25/03 & 09/04	☐	B	S	L	Tx	SPC
Pattawa (dog)	after 15/05	☐	B	S	L	Tx	SPC
Cabiai (dog)		☐	B	S	L	Tx	SPC
Mother cat		☐	B	S	L	Tx	SPC
Ginger cat		☐	B	S	L	Tx	SPC
Black cat		☐	B	S	L	Tx	SPC
Other:	Where:	☐	B	S	L	Tx	SPC
Other:	Where:	☐	B	S	L	Tx	SPC

[1]B: bite; S: scratch; L: Licked mucosa/skin abrasion; Tx: treatment; SPC: simple physical contact (e.g. petting).

Were you in contact with the case of human rabies since 1 May? Yes ☐ No ☐

Were you in contact with the human rabies case during his illness? Yes ☐ No ☐

Do you know of other animals that had been in contact with the human rabies case before his illness? Yes ☐ No ☐

If Yes, what animals?

Type: ....
Date of contact:.............................
Type of contact:..............................
Site of contact: ....
Family name/first name of owner:
....
Contact information:..........................
Current status of the animal:

Type: ....
Date of contact:.............................
Type of contact:..............................
Site of contact:...... ....
Family name/first name of owner:
....
Contact information:............................
Current status of the animal:

Type:...
Date of contact:.............................
Type of contact:..............................
Site of contact: ....
Family name/first name of owner:
....
Contact information:............................
Current status of the animal:

Do you know of other individuals who have been in contact with the human rabies case during his illness? (Who might not be aware of his illness)? Yes ☐ No ☐

Family name/first name: .... Contact information: ....
Family name/first name: .... Contact information: ....
Family name/first name: .... Contact information: ....
Family name/first name: .... Contact information: ....

Anti-rabies consultation? Yes ☐ No ☐ Date ___/_____/__________ Where: ....

Vaccination Yes ☐ No ☐ D0: __/____. JD7: ___/_____ D21: ___/______

Supplementary information:

....
